# A Systematic Review of Deep Learning Approaches for Hepatopancreatic Tumor Segmentation

**DOI:** 10.3390/jimaging12040147

**Published:** 2026-03-26

**Authors:** Razeen Hussain, Muhammad Mohsin, Dadan Khan, Mohammad Zohaib

**Affiliations:** Department of Informatics, Bioengineering, Robotics and Systems Engineering, University of Genoa, 16146 Genoa, Italy; dadan.khan@edu.unige.it (D.K.); mohammad.zohaib@edu.unige.it (M.Z.)

**Keywords:** liver, pancreas, hepatopancreatic, tumor, CT, MRI, segmentation, deep learning

## Abstract

Deep learning has advanced rapidly in medical image segmentation, yet hepatopancreatic tumor delineation remains challenging due to low contrast, small lesion size, organ variability, and limited high-quality annotations. Existing reviews are outdated or overly broad, leaving recent architectural developments, training strategies, and dataset limitations insufficiently synthesized. To address this gap, we conducted a PRISMA 2020 systematic literature review of studies published between 2021 and 2026 on deep learning-based liver and pancreatic tumor segmentation. From 2307 records, 84 studies met inclusion criteria. U-Net variants continue to dominate, achieving strong liver segmentation but inconsistent tumor accuracy, while transformer-based and hybrid models improve global context modeling at higher computational cost. Attention mechanisms, boundary-refinement modules, and semi-supervised learning offer incremental gains, yet pancreatic tumor segmentation remains notably difficult. Persistent issues, including domain shift, class imbalance, and limited generalization across datasets, underscore the need for more robust architectures, standardized benchmarks, and clinically oriented evaluation. This review consolidates recent progress and highlights key challenges that must be addressed to advance reliable hepatopancreatic tumor segmentation.

## 1. Introduction

Hepatopancreatic tumors, encompassing primary and secondary malignancies of the liver and pancreas, remain among the most lethal and clinically challenging cancers worldwide [1]. Hepatocellular carcinoma (HCC) is the sixth most commonly diagnosed cancer and the third leading cause of cancer-related mortality [2], while pancreatic ductal adenocarcinoma (PDAC) continues to exhibit one of the lowest five-year survival rates among all solid tumors due to its aggressive biology and late clinical presentation [3]. Both disease groups are characterized by complex tumor–organ interactions, high vascularization, and anatomical proximity to critical structures, making accurate assessment essential for clinical decision-making across diagnosis, staging, surgical planning, and treatment monitoring [4].

Medical imaging plays a central role in the management of hepatopancreatic malignancies [5,6]. Contrast-enhanced CT and MRI remain the primary modalities for tumor detection, characterization, and longitudinal follow-up, while PET imaging provides complementary metabolic information in selected cases. However, the reliable delineation of tumors and surrounding anatomy from these modalities is non-trivial. Lesions often present with heterogeneous enhancement patterns, irregular shapes, and low contrast relative to adjacent parenchyma. Pancreatic tumors, in particular, may be isoattenuating on CT, while liver lesions can exhibit variable arterial and venous phase behavior depending on vascular supply. These factors contribute to substantial inter- and intra-observer variability in manual segmentation, which is time-consuming and difficult to standardize across institutions and imaging protocols [7].

Automated segmentation methods therefore hold significant promise for improving reproducibility, reducing clinical workload, and enabling quantitative imaging biomarkers. Yet, hepatopancreatic tumor segmentation remains one of the most technically challenging tasks in medical image analysis [8,9]. Tumors may be small, infiltrative, or poorly demarcated; imaging artifacts such as respiratory motion, partial-volume effects, and modality-specific noise further complicate boundary identification [10]. Anatomical variability across patients, differences in acquisition protocols, and domain shifts between scanners or institutions introduce additional sources of error. Multimodal imaging integration, while potentially beneficial, adds complexity due to heterogeneous spatial resolution, contrast behavior, and alignment requirements.

Over the past decade, deep learning (DL) has transformed medical image segmentation [11], with convolutional neural networks (CNNs) and encoder–decoder architectures such as U-Net [12] becoming the dominant paradigm. Recent methodological advances have introduced attention mechanisms, multi-scale feature fusion, transformer-based architectures, and hybrid CNN–transformer models that improve long-range dependency modeling and contextual reasoning. Parallel developments in semi-supervised learning, domain adaptation, and uncertainty estimation have further expanded the applicability of DL models to heterogeneous clinical datasets. In hepatopancreatic imaging specifically, these innovations have enabled more robust tumor delineation, improved handling of low-contrast lesions, and better integration of multiphase or multimodal data.

From a clinical perspective, the need for reliable automated segmentation is further amplified by the increasing adoption of image-guided interventions, radiomics, and personalized treatment planning [13,14,15]. Liver and pancreatic cancer management increasingly relies on quantitative imaging biomarkers for assessing tumor burden, vascular involvement, resectability, and treatment response. Radiomics pipelines, for example, require consistent and reproducible segmentations to extract meaningful features. Similarly, surgical planning systems and intraoperative navigation platforms depend on accurate 3D models of tumors and surrounding anatomy. As these technologies move toward routine clinical use, the demand for robust, generalizable segmentation algorithms continues to grow.

At the same time, the research landscape has become more complex due to the proliferation of datasets, evaluation protocols, and architectural variants. Public datasets such as LiTS, MSD, and Pancreas-CT have catalyzed progress but differ substantially in imaging characteristics, annotation quality, and task definitions. Many studies report results on small or institution-specific datasets, making cross-study comparison difficult. Furthermore, the rapid emergence of transformer-based models, hybrid architectures, and self-supervised pretraining strategies has created a methodological landscape that is both rich and fragmented. A systematic synthesis of these developments is essential for understanding which innovations meaningfully advance the state of the art and which challenges remain unresolved.

Despite substantial growth in the field, existing reviews [16,17,18] either provide broad overviews of abdominal organ segmentation or focus on studies published before 2021. For example, earlier reviews largely examine classical image analysis methods or early deep learning models, and therefore, do not capture recent advances such as hybrid CNN architectures, attention mechanisms, transformer-based networks, and semi-supervised learning strategies that have significantly improved tumor segmentation performance. In addition, many prior surveys focus mainly on liver segmentation or general abdominal imaging, without providing a detailed comparison of both liver and pancreas tumor segmentation approaches, datasets, and evaluation protocols.

To address this gap, This systematic literature review analyzes deep learning approaches for hepatopancreatic tumor segmentation published from 2021 onward, examining network architectures, imaging modalities, datasets, evaluation metrics, and reported performance. By synthesizing recent evidence, it aims to clarify the current state of the art, support methodological comparison and reproducibility, and outline key directions for future research and clinical translation.

## 2. Methodology

This systematic literature review follows the PRISMA 2020 guidelines [19] to ensure transparency, reproducibility, and methodological rigor in the identification, screening, and analysis of relevant studies. The review focuses on deep learning-based computer vision methods for hepatopancreatic tumor segmentation published from 2021 onwards.

This review was not prospectively registered in the PROSPERO database. Although protocol registration is recommended to enhance transparency, the review followed a predefined search and study selection strategy. The search strategy was structured using the PICO(S) framework (Population, Intervention, Comparison, Outcomes, and Study design) to define the scope of the review and guide the selection of search terms. The detailed PICO(S) specification is provided in Appendix A.1.

### 2.1. Research Questions

The review protocol was designed to address recent advances in deep learning for segmentation, with an emphasis on algorithmic design, datasets, and evaluation practices. The following research questions (RQs) guide the review:

- RQ1: What imaging modalities and datasets are available for hepatopancreatic tumor segmentation?
- RQ2: Which deep learning methods and evaulation metrics have been used for hepatopancreatic tumor segmentation?
- RQ3: What organ-specific challenges can be addressed by the deep learning community?

### 2.2. Search Criteria

A comprehensive literature search was conducted on 8 January 2026 using the Scopus, IEEE Xplore, PubMed and Web of Science digital libraries, as they collectively cover a broad spectrum of computer vision, medical imaging, and deep learning research published in journals and conference proceedings.

The following search query was used consistently across databases, with database-specific syntax adaptations where necessary: “deep learning” AND (“liver” OR “pancreas”) AND “segmentation” AND (“tumor” OR “tumors” OR “tumour” OR “tumours”).

This query was designed to capture studies explicitly addressing deep learning-based segmentation of liver and pancreatic tumors while excluding non-segmentation and non-deep learning work. For databases such as PubMed, relevant controlled vocabulary terms (e.g., MeSH) were also considered. We iteratively validated the search query by verifying that well-known benchmark studies were retrieved and made minor adjustments to balance search sensitivity and specificity. Although related terminology such as lesion, neoplasm, mass, or cancer may appear in the radiology literature, the query intentionally focused on the term tumor to prioritize studies explicitly addressing tumor segmentation and to reduce the retrieval of unrelated tasks such as lesion detection, classification, or radiomic analysis.

The initial search returned 849 papers from Scopus, 563 papers from IEEE Xplore, 274 papers from PubMed and 621 papers from Web of Science resulting in a total of 2307 records prior to deduplication.

### 2.3. Selection Process

The study selection process was conducted in multiple stages in accordance with PRISMA 2020 recommendations:

- Deduplication: Duplicate records across databases were identified and removed.
- Title and Abstract Screening: Remaining studies were screened based on relevance to deep learning, segmentation, and hepatopancreatic organs.
- Full-Text Review: Shortlisted studies underwent full-text evaluation against the inclusion and exclusion criteria.

**Figure 1:**
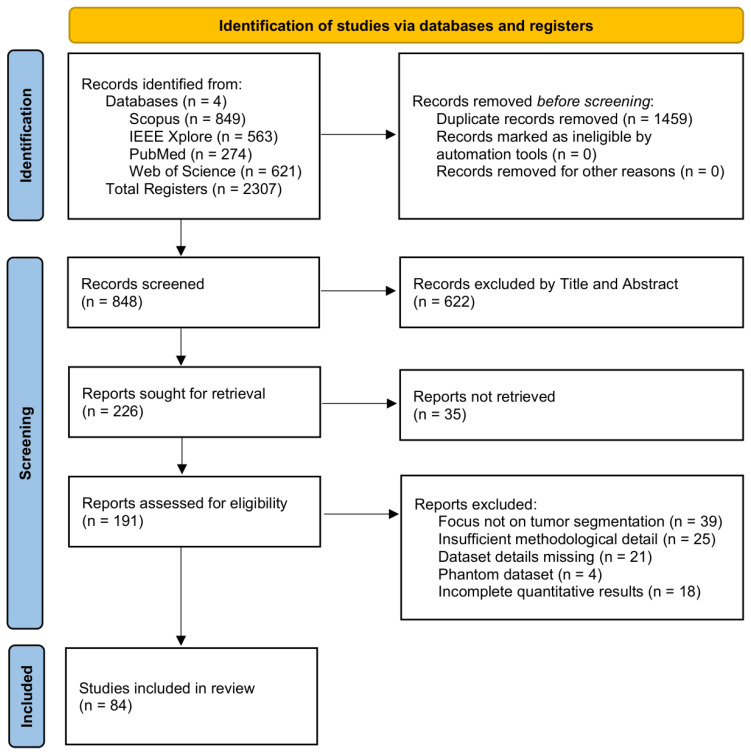
PRISMA 2020 flow diagram summarizing the studies selection process.

Following title and abstract screening, 226 papers were shortlisted for detailed assessment. After full-text review, 84 studies were finalized for inclusion in the systematic review. Please see Appendix A.2 for the list of included studies. The selection process is summarized in the PRISMA flow diagram illustrated in Figure 1.

### 2.4. Inclusion Criteria

Studies were included if they satisfied all of the following conditions:

- Peer-reviewed journal articles or conference papers.
- Studies that apply deep learning methods.
- Studies focused on liver and/or pancreas.
- Studies reporting at least one quantitative evaluation metric.
- Studies published between 2021–2026.
- Studies written in English.
- Multi-organ segmentation papers if they report organ-specific results for at least one of the two organs.

### 2.5. Exclusion Criteria

Studies were excluded based on the following criteria:

- Studies that do not use deep learning (e.g., classical ML, radiomics-only, manual segmentation).
- Studies focusing on other organs (brain, lung, heart, kidney, breast, musculoskeletal), unless liver and/or pancreas are explicitly analyzed.
- Studies performing tasks other than segmentation such as image reconstruction, enhancement, registration, denoising, tracking, and simulation.
- Studies without quantitative results or insufficient methodological detail.
- Animal studies, phantom studies, or synthetic-only datasets (unless real human imaging is included).
- Duplicate publications or extended versions of the same study without new deep learning results.
- Review papers, systematic reviews, meta-analyses, editorials, letters, or abstracts without full text.

### 2.6. Data Extraction and Synthesis

The study selection and data extraction processes were conducted by four independent reviewers to ensure methodological rigor and reduce selection bias. Initially, all four reviewers collaboratively defined the research questions as well as the inclusion and exclusion criteria. The title and abstract screening stage was performed collectively during a joint meeting to ensure a consistent interpretation of the eligibility criteria.

Following this stage, 191 studies remained for full-text evaluation. These studies were distributed among the reviewers for independent screening based on the predefined criteria. Any uncertainties or disagreements regarding study eligibility were resolved through discussion and consensus among the four reviewers. After finalizing the list of included studies, the reviewers jointly determined the variables to be extracted from each paper.

For the data extraction stage, the final set of 84 studies was evenly distributed among the four reviewers. Each reviewer independently extracted the predefined information from their assigned papers. Once the extraction process was completed, the reviewers conducted a joint meeting to standardize and verify the extracted data, ensuring consistency across all studies included in the review.

For each included study, relevant information was systematically extracted, including imaging modality, dataset characteristics, network architecture, evaluation metrics, and reported performance. Studies were analyzed and synthesized qualitatively, with comparative discussion structured around the defined research questions.

Rather than performing a meta-analysis, which is challenging due to dataset heterogeneity and inconsistent evaluation protocols, this review emphasizes methodological trends, architectural design choices, and recurring challenges specific to liver and pancreatic tumor segmentation from a computer vision standpoint.

**Figure 2:**
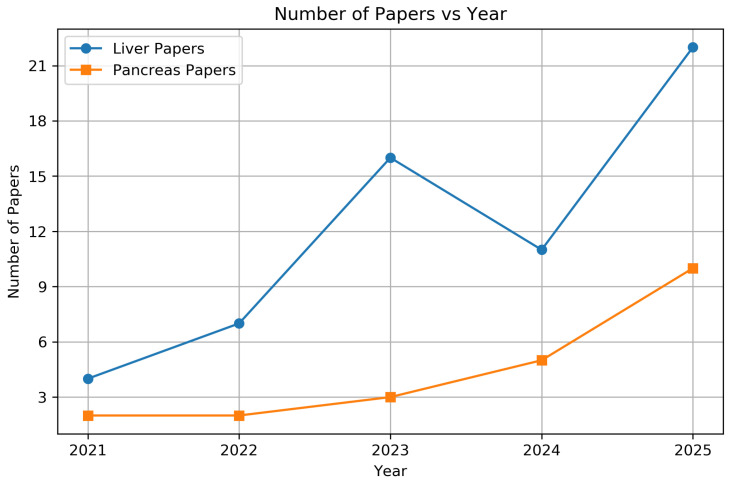
Plot highlighting the growing interest in liver and pancreas tumor segmentation.

Figure 2 highlights the increasing research interest in liver and pancreas tumor segmentation from 2021 to 2025. Liver-focused studies show a marked rise, particularly after 2022, while pancreas-related publications also demonstrate steady growth. This upward trend reflects the expanding attention to hepatopancreatic tumor segmentation within the deep learning community.

## 3. Datasets

**Figure 3:**
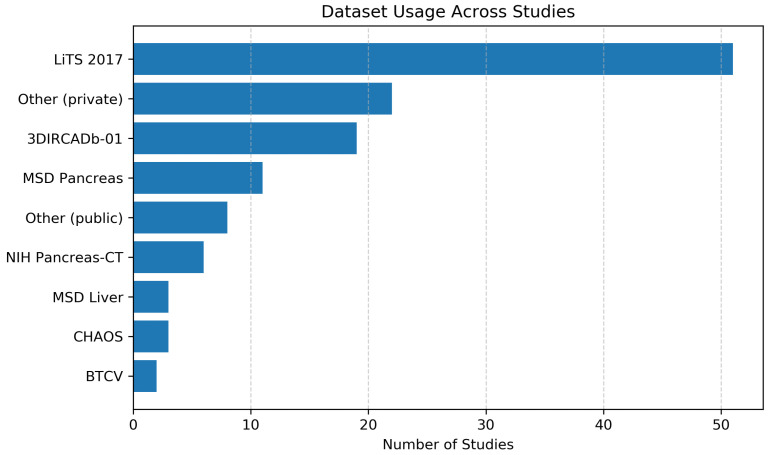
Distribution of datasets used across the included studies [20,72,77,89,93,94].

Publicly available datasets play a central role in benchmarking deep learning models for hepatopancreatic tumor segmentation. From a computer vision perspective, dataset characteristics, such as imaging modality, annotation granularity, class distribution, and sample size, directly influence architectural design choices, training strategies, and evaluation protocols. The reviewed studies primarily rely on CT-based datasets, with comparatively fewer MRI-based or multi-modal resources. The list of publicly available datasets for liver and/or pancreas segmentation can be seen in Table 1, while Figure 3 shows the distribution of datasets used across the included studies.

### 3.1. Liver Segmentation Datasets

The most widely used benchmark is LiTS 2017 [20], which contains 201 contrast-enhanced CT volumes with annotations for both liver and tumors. Its binary annotation structure (liver vs. tumor) and relatively large cohort have made it the de-facto standard for liver tumor segmentation benchmarking. As shown in Figure 3, which summarizes dataset usage across the reviewed literature, 51 out of the 84 included studies utilize the LiTS dataset. Given that 62 studies in this review focus on liver tumor segmentation, LiTS represents the most frequently adopted dataset, making it central to comparative performance analysis.

Another frequently used CT dataset is 3DIRCADb-01 [72], comprising 22 cases with detailed annotations for abdominal organs and liver tumors. Although smaller in size, its fine-grained labels and complex tumor morphology make it suitable for validation and cross-dataset generalization studies.

CHAOS [93] dataset provides both CT and MRI scans (40 cases) with multi-organ annotations. While originally designed for abdominal organ segmentation, it is occasionally used to evaluate liver segmentation performance in multi-modal settings.

MRI-focused liver datasets are comparatively limited. The Duke Liver Dataset (DLDS) [95] dataset includes 105 MRI cases and supports both classification and segmentation tasks. Similarly, ATLAS [97] provides 90 multi-phase, multi-center CT and MRI cases with liver and hepatocellular carcinoma (HCC) annotations, enabling evaluation of multi-phase fusion and domain generalization methods.

### 3.2. Pancreas Segmentation Datasets

Pancreatic tumor segmentation is more challenging due to organ size, shape variability, and low contrast in CT images. The largest and most widely adopted dataset is MSD Pancreas [77], which includes 420 CT cases annotated for pancreas and tumor regions. Its scale and tumor annotations make it a primary benchmark for pancreas tumor segmentation.

The NIH Pancreas-CT dataset [89] contains 82 CT scans with pancreas annotations. Although it primarily includes healthy pancreas masks (single-category annotation), it is frequently used for pretraining, organ localization, or auxiliary segmentation tasks in multi-stage frameworks.

### 3.3. Multiple Organ Datasets

Several datasets include both liver and pancreas annotations within broader abdominal segmentation benchmarks, such as BTCV [94] (50 CT cases) with 13 abdominal organ labels, FLARE 2021 [101] (511 CT cases) featuring four abdominal organ categories and AbdomenAtlas Mini [102] (5195 CT cases), one of the largest abdominal CT datasets, containing nine organ categories including liver and pancreas. Although these datasets are not tumor-specific, they are commonly used for organ localization, pretraining, multi-task learning, and transfer learning strategies before fine-tuning on tumor segmentation datasets.

## 4. Evaluation Metrics

**Figure 4:**
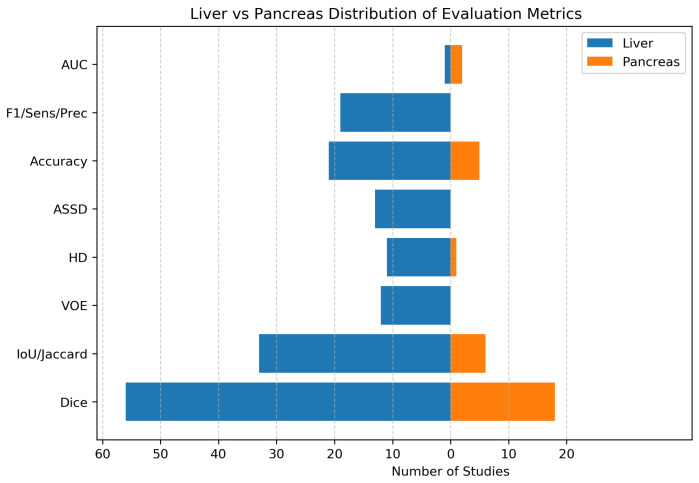
Distribution of evaluation metrics reported in liver and pancreas studies.

In the included studies, various performance metrics were employed to evaluate the efficacy of the models, particularly in the context of tumor segmentation. These metrics are broadly categorized into overlap-based, distance-based, and statistical measures, providing a comprehensive assessment of model segmentation performance. The interpretation of these metrics often relies on the concepts of True Positives (TP), False Positives (FP), True Negatives (TN), and False Negatives (FN), which quantify the correctness of pixel-wise classifications. The selection of appropriate metrics is crucial for accurately reflecting the clinical relevance and technical performance of segmentation models. Table 2 summarizes the metrics and their primary focus in the context of this review, highlighting their relevance to tumor segmentation while Figure 4 illustrates the frequency of usage of evaluation metrics across liver and pancreas tumor segmentation studies.

### 4.1. Overlap-Based Metrics

Overlap-based metrics are best understood through the concept of Venn diagrams, illustrating the relationship between the predicted positive region (*P*) and the actual positive region (*G*). In the context of binary segmentation, these regions can be further broken down into TP, FP, and FN. A visual representation would typically show two overlapping circles, one for *P* and one for *G*, where the overlap represents TP=P∩G, the part of *P* not overlapping *G* represents FP=P∖G, and the part of *G* not overlapping *P* represents FN=G∖P. The union (P∪G) then corresponds to TP+FP+FN. These metrics are fundamental for assessing how well a segmentation model delineates the region of interest, directly relating to the counts of TP, FP, and FN.

#### 4.1.1. Dice Similarity Coefficient

The Dice Similarity Coefficient (DSC), often referred to as the F1-score in the context of binary segmentation, is the most widely used metric in hepatopancreatic tumor segmentation due to its intuitive interpretation and robustness. It measures the spatial overlap between the *P* and *G*. In terms of TP, FP, and FN, DSC is defined as:

$$DSC=\frac{2\times TP}{2\times TP+FP+FN}$$

where TP represents the number of pixels correctly identified as positive, FP represents pixels incorrectly identified as positive, and FN represents pixels incorrectly identified as negative. The DSC value ranges from 0 to 1, where 1 indicates perfect overlap and 0 signifies no overlap. A higher DSC value indicates better segmentation performance. The DSC is particularly sensitive to small segmentation errors and is often preferred when both FP and FN are equally important.

#### 4.1.2. Jaccard Index and Intersection over Union

The Jaccard Index, commonly known as Intersection over Union (IoU), is another standard metric for evaluating tumor segmentation accuracy. It calculates the ratio of the intersection of *P* and *G* to their union. In terms of TP, FP, and FN, IoU is defined as:

$$Jaccard=IoU=\frac{TP}{TP+FP+FN}$$

Similar to DSC, the IoU also ranges from 0 to 1, with 1 indicating perfect overlap. While DSC and IoU are closely related (DSC = 2×IoU/(1+IoU)), IoU tends to penalize under- and over-segmentation more heavily than DSC, making it more sensitive to single instances of misclassification. This characteristic makes IoU a stricter metric, often resulting in lower values compared to DSC for the same segmentation result.

#### 4.1.3. Volumetric Overlap Error

Volumetric Overlap Error (VOE) is a metric that quantifies the disagreement between the predicted positive region and the actual positive region. It represents the complement of the Jaccard Index and is frequently reported in volumetric segmentation tasks. In terms of TP, FP, and FN, VOE is defined as:

$$VOE=1-\frac{TP}{TP+FP+FN}$$

Often, VOE is expressed as a percentage: $VOE=100\times \left(1-\frac{TP}{TP+FP+FN}\right)$. A VOE of 0% indicates a perfect segmentation (i.e., complete agreement between the predicted and actual positive regions), while 100% indicates no overlap whatsoever. Lower VOE values signify better segmentation performance, as they imply less error in volumetric estimation.

### 4.2. Distance-Based Metrics

Distance-based metrics focus on the boundaries of the segmented regions. These boundaries implicitly define the actual positive (foreground) and negative (background) regions. A visualization for these metrics would typically depict two irregular shapes, representing the boundary of the predicted positive region and the boundary of the actual positive region, and illustrate the shortest distances between points on these boundaries. These metrics are particularly critical for clinical applications where the accurate delineation of object boundaries is paramount, such as in surgical planning or radiation therapy, where even small deviations can have significant consequences.

#### 4.2.1. Hausdorff Distance

The Hausdorff Distance (HD) is a metric that measures the maximum distance between the boundary of the predicted positive region (∂P) and the boundary of the actual positive region (∂G). It is defined as:

$$HD\left(P,G\right)=\max\left(\underset{p\in \partial P}{\sup}\underset{g\in \partial G}{\inf}∥p-g∥,\underset{g\in \partial G}{\sup}\underset{p\in \partial P}{\inf}∥g-p∥\right)$$

where sup denotes the supremum (least upper bound), inf denotes the infimum (greatest lower bound), and ∥p−g∥ represents the Euclidean distance between points *p* and *g*. The HD essentially finds the point on one boundary that is farthest from the other boundary, and vice versa, taking the maximum of these two values. A lower HD value indicates better boundary agreement.

However, the standard HD is highly sensitive to outliers and small noise artifacts, as a single misclassified voxel far from the true boundary can significantly inflate the HD value. To mitigate this sensitivity, the HD95 is often preferred. HD95 calculates the 95th percentile of the distances between boundary points rather than the maximum. This makes it more robust to extreme outliers while still providing a strong indication of boundary accuracy. Both HD and HD95 are measured in units of distance (e.g., millimeters or pixels).

#### 4.2.2. Average Symmetric Surface Distance

The Average Symmetric Surface Distance (ASSD) computes the mean distance between the surfaces of the predicted segmentation and the ground truth in both directions. It evaluates the average boundary discrepancy and is less sensitive to outliers than the Hausdorff Distance. ASSD is defined as:

(1) $$ASSD\left(A,B\right)=\frac{1}{\left|S_{A}|+|S_{B}\right|}\left(\sum_{a\in S_{A}}d\left(a,S_{B}\right)+\sum_{b\in S_{B}}d\left(b,S_{A}\right)\right)$$

where $S_{A}$ and $S_{B}$ denote the sets of surface voxels (or boundary points) of the predicted mask *A* and ground truth mask *B*, respectively, and $d(a,S_{B})$ represents the minimum Euclidean distance from point *a* to the surface $S_{B}$.

### 4.3. Statistical and Classification Metrics

Statistical and classification metrics are often visualized using a confusion matrix or a Receiver Operating Characteristic (ROC) curve. A confusion matrix is a table that summarizes the performance of a classification algorithm, showing the counts of TP, TN, FP, and FN. An ROC curve, on the other hand, graphically illustrates the diagnostic ability of a binary classifier system as its discrimination threshold is varied. It plots the True Positive Rate (Sensitivity) against the False Positive Rate (1–Specificity) at various threshold settings, with the Area Under the Curve (AUC) providing a single performance measure. These metrics evaluate the performance based on the pixel-wise classification of the image into foreground (region of interest) and background.

#### 4.3.1. Accuracy

Accuracy measures the proportion of total pixels that were correctly classified, encompassing both TP and TN. It is defined as:

$$\mathrm{Accuracy}=\frac{TP+TN}{TP+TN+FP+FN}$$

While intuitive, accuracy can be misleading in scenarios with highly imbalanced datasets, where the majority class dominates the calculation. For instance, in tumor detection, if the region of interest is very small compared to the background, a high accuracy can be achieved by simply classifying all pixels as background, even if the actual region of interest is completely missed.

#### 4.3.2. Precision, Sensitivity, and F1-Score

These metrics provide a more nuanced view of classification performance, especially in imbalanced datasets:

- Precision: Precision, also known as the Positive Predictive Value, measures the proportion of correctly predicted positive pixels out of all pixels predicted as positive. It quantifies the reliability of positive predictions.
  $$\mathrm{Precision}=\frac{TP}{TP+FP}$$
- Sensitivity: Sensitivity, also known as Recall or True Positive Rate, measures the proportion of correctly predicted positive pixels out of all actual positive pixels in the ground truth. It quantifies the model’s ability to detect all positive instances.
  $$\mathrm{Sensitivity}=\frac{TP}{TP+FN}$$
- F1-Score: The F1-Score is the harmonic mean of Precision and Sensitivity. It provides a balanced measure that considers both FP and FN, making it particularly useful when there is an uneven class distribution or when both precision and recall are important.
  $$F1=2\times \frac{\mathrm{Precision}\times \mathrm{Sensitivity}}{\mathrm{Precision}+\mathrm{Sensitivity}}$$

#### 4.3.3. Area Under the Receiver Operating Characteristic Curve

The AUC is a robust metric for evaluating the overall discriminative power of a binary classifier across all possible classification thresholds. The ROC curve plots the True Positive Rate (Sensitivity) against the False Positive Rate (1–Specificity) at various threshold settings. Specificity is defined as:

$$\mathrm{Specificity}=\frac{TN}{TN+FP}.$$

An AUC value ranges from 0 to 1.0. An AUC of 1.0 represents a perfect model that can distinguish between positive and negative classes without error, while an AUC of 0.5 suggests a model no better than random chance. AUC is particularly valuable because it is insensitive to class imbalance and provides a single scalar value that summarizes the model’s performance across all possible operating points.

While AUC is generally robust to moderate class imbalance, it can overestimate model performance in extreme prevalence scenarios because it gives equal weight to all thresholds regardless of class distribution. Therefore, complementary metrics such as precision, recall, and F1-score are usually reported as well to provide a more complete evaluation, especially for highly imbalanced datasets.

## 5. Tumor Segmentation Models

From a computer vision standpoint, the hepatopancreatic tumor segmentation presents a hierarchical structure:

1. Organ extraction from surrounding abdominal structures;
2. Tumor delineation.

### 5.1. Liver Tumor Segmentation

Liver tumor segmentation has been one of the most extensively studied problems in abdominal medical image segmentation, particularly in CT imaging. While liver segmentation has largely reached performance saturation on benchmark datasets, tumor segmentation remains significantly more challenging due to small lesion size, irregular morphology, low contrast, and severe class imbalance. Various approaches related to liver tumor segmentation are summarized in Table 3.

#### 5.1.1. U-Net and Its Variants

U-Net [12] and its architectural derivatives remain the dominant baseline for liver tumor segmentation and are still the most frequently adopted reference architecture in studies published after 2021. On standard CT benchmarks, U-Net–based models consistently report liver Dice scores in the range of 0.96–0.97 [38,42,61,69], indicating that whole-liver segmentation has reached near-saturation under controlled experimental conditions. However, this performance does not directly translate to tumor segmentation [21,28], where Dice scores are substantially lower due to heterogeneous appearance, irregular morphology, diffuse boundaries, and extreme class imbalance.

The continued popularity of U-Net stems from its effective encoder–decoder design with skip connections that transfer high-resolution spatial features from early layers to deeper decoding stages. This structure enables precise boundary delineation and strong multi-scale feature aggregation while maintaining relatively low architectural complexity. U-Net variants are also highly adaptable, supporting 2D, 2.5D, and 3D implementations, residual backbones, dense connections, and lightweight modifications [28]. Importantly, they converge reliably even on modest-sized medical imaging datasets [21,23,32,48,76], making them practical for scenarios where annotated data are limited.

Despite these strengths, U-Net’s inductive bias toward local convolutional operations constrains its ability to model long-range spatial dependencies. This limitation becomes particularly evident in tumor segmentation, where contextual reasoning across distant regions may help distinguish lesions from vessels, artifacts, or normal parenchyma. Very small, low-contrast, deformable, or irregularly shaped tumors are frequently under-segmented or entirely missed, especially when their volumetric footprint is negligible relative to the liver. Furthermore, U-Net variants often exhibit reduced robustness under cross-dataset evaluation, highlighting sensitivity to domain shift and acquisition variability.

#### 5.1.2. Encoder–Decoder Architectures Beyond U-Net

Encoder–decoder architectures that extend beyond the original U-Net typically incorporate stronger backbone networks, such as residual or densely connected encoders, to enhance feature extraction capacity. These models generally report liver Dice scores up to 0.95 [24,56], performing competitively but not substantially surpassing well-optimized U-Net variants. The primary contribution of these architectures lies in improved representation depth and feature reuse rather than in a fundamentally different segmentation paradigm.

In practice, deeper or more expressive encoders can produce sharper and less coarse segmentation masks, which is particularly relevant in applications where boundary precision influences downstream analysis or surgical planning [49]. However, these gains are often incremental and primarily affect organ-level segmentation rather than tumor-specific accuracy [28,100]. Because these models remain convolution-dominated, their receptive fields are still governed by local spatial aggregation, limiting their ability to capture long-range anatomical dependencies or contextual relationships across the liver volume.

As with U-Net, small or low-contrast tumors remain difficult to segment reliably [37,40]. Performance degrades in cases of subtle intensity variation, motion artifacts, or heterogeneous enhancement patterns in CT and MRI scans. Although richer encoders may improve robustness to some noise patterns, they do not fundamentally address class imbalance or the sparse nature of tumor voxels. The modest performance gap between these architectures and standard U-Net variants suggests that increasing backbone complexity alone is insufficient to overcome the intrinsic challenges of liver tumor segmentation. Consequently, improvements achieved by encoder substitution often reflect engineering refinement rather than a structural solution to tumor-specific modeling limitations.

#### 5.1.3. Transformer-Based Models

Transformer-based segmentation models have emerged as an alternative to purely convolutional architectures by explicitly modeling long-range spatial dependencies through self-attention mechanisms. Unlike standard CNNs or U-Net variants, which rely primarily on local receptive fields, transformers relate distant regions within the image volume, theoretically enabling improved tumor localization and contextual reasoning. In liver tumor segmentation, reported tumor Dice scores for transformer-based approaches are commonly around 0.80 [34,50], with improvements in VOE compared to some convolution-only baselines.

The principal advantage of transformer-based models lies in global context integration. By allowing each token or patch to attend to all others, these architectures can better capture spatially distant yet semantically related regions, which is particularly relevant for irregularly shaped or infiltrative tumors. In theory, this capability supports more coherent volumetric predictions and reduces fragmented segmentations.

However, the empirical gains are often less dramatic than the theoretical promise suggests. Transformer models introduce substantial computational complexity and higher memory requirements, especially in 3D medical imaging contexts [58,96]. Their performance is also highly dependent on dataset scale; without sufficient training data, self-attention mechanisms may overfit or fail to generalize effectively. In many studies, improvements over strong CNN baselines are incremental rather than transformative, raising questions about cost–benefit trade-offs in resource-constrained environments.

Hybrid CNN–Transformer architectures attempt to balance the inductive bias of convolutions with the global reasoning capacity of attention mechanisms [34]. By preserving local feature extraction in early layers and introducing attention modules at deeper stages, these models aim to mitigate the data inefficiency of pure transformers. While hybrids represent a growing trend and often achieve competitive performance, their architectural complexity increases substantially, and reproducibility across datasets remains inconsistent.

Vision Transformer-based encoder frameworks, such as UNETR-style designs [107], have shown particular promise for small lesion segmentation, where global contextual reasoning may help differentiate subtle tumor regions from surrounding parenchyma [58]. Nevertheless, these models typically require large-scale datasets and significant computational resources to realize their potential. In data-scarce or heterogeneous settings (common in liver tumor segmentation) their advantages may diminish, suggesting that transformer-based approaches are not universally superior but rather context-dependent solutions whose benefits must be evaluated against practical constraints.

#### 5.1.4. Optimization-Based and Boundary Refinement Methods

Optimization-based and boundary refinement approaches aim to improve tumor delineation by refining initial segmentation outputs through post-processing modules or clinically inspired constraints. These methods [22,45,51] often enhance boundary precision and reduce surface distance errors, reporting tumor Dice scores in the range of 0.80–0.92 along with lower ASSD. In many cases, they employ relatively lightweight refinement architectures that improve contour accuracy without dramatically increasing model size, helping to limit overfitting [63].

Despite these advantages, their effectiveness depends heavily on the quality of the initial segmentation [51,57,100]. If the base prediction is poor, particularly for small or low-contrast tumors, refinement stages offer limited corrective capacity. Moreover, optimized or fine-tuned architectures remain sensitive to dataset size and diversity, and performance gains may not transfer well across domains. Hyperparameter tuning and architectural optimization also require substantial expertise and experimentation, which can reduce reproducibility and practical scalability.

#### 5.1.5. Attention-Based Networks

Attention mechanisms, including channel attention, spatial attention, and hybrid attention modules, are frequently incorporated into U-Net-like architectures to refine feature selection and improve tumor localization. By reweighting feature maps or spatial regions, these models aim to emphasize tumor-relevant structures while suppressing background noise. In liver tumor segmentation, attention-enhanced networks often report improved performance compared to plain encoder–decoder baselines, particularly for small lesions and boundary-sensitive cases [25,26,32,61].

The practical appeal of attention modules lies in their ability to guide the network toward salient regions without fundamentally altering the overall architecture. They are especially effective in capturing complex tumor shapes and improving delineation in heterogeneous or noisy CT scans [31,46,70]. In many cases, attention helps reduce false positives by discouraging activation in anatomically implausible regions, thereby improving qualitative consistency.

However, the performance gains are frequently incremental rather than paradigm-shifting [33,38,39,62]. Attention mechanisms increase architectural complexity, parameter count, and computational overhead, and their effectiveness depends heavily on careful integration within the backbone network. Poorly designed attention modules can introduce instability or redundant feature weighting without meaningful improvement. Moreover, attention does not inherently resolve core challenges such as severe class imbalance or cross-dataset generalization. When training data are limited, additional parameters may even increase the risk of overfitting [53]. Thus, while attention-based CNNs represent one of the most practical and widely adopted strategies for incremental performance enhancement in liver tumor segmentation, they should be viewed as refinement mechanisms rather than standalone solutions to the fundamental difficulties of tumor modeling.

#### 5.1.6. Mask R-CNN and Detection–Segmentation Hybrids

Region-based frameworks such as Mask R-CNN have been adapted for liver tumor analysis by jointly performing lesion detection and mask prediction. Conceptually, these models are attractive when both tumor localization and segmentation are required, particularly in cases involving multiple or spatially separated lesions. The detection stage can help restrict segmentation to plausible regions, potentially reducing false positives [44,75].

In practice, however, Mask R-CNN–style approaches generally underperform compared to dedicated dense segmentation networks in volumetric CT settings [52,64]. Instance-based detection frameworks are not naturally suited to continuous, irregular tumor masks that span multiple slices. Moreover, the two-stage pipeline and additional mask head increase memory usage and inference time, which becomes problematic for high-resolution 3D scans. As a result, while detection–segmentation hybrids provide a unified framework, they rarely surpass optimized encoder–decoder models in pixel-level accuracy.

#### 5.1.7. Graph CNNs and Structural Modeling

Graph convolutional networks (GCNs) have been explored to model relational and structural information within liver and tumor regions [43,51]. By representing image elements as nodes connected through edges, these methods capture non-Euclidean relationships and can explicitly encode spatial dependencies beyond regular grid structures. This property makes them theoretically well suited for modeling complex tumor shapes and irregular boundaries.

Empirically, graph-based models report competitive Dice and IoU scores, along with balanced sensitivity and specificity [39,43]. However, constructing graphs from volumetric images introduces significant computational overhead. The conversion from voxel grids to graph representations increases preprocessing complexity and memory demands. Furthermore, the added architectural sophistication does not consistently translate into large performance gains over strong CNN baselines. While structurally appealing, GCN-based approaches remain relatively niche due to their implementation complexity and scalability concerns.

#### 5.1.8. Hybrid CNN Models

Hybrid CNN architectures, combining residual backbones, attention modules, multi-scale fusion strategies, or transformer components, have become a dominant trend in recent years [27,29,30]. These models aim to integrate complementary inductive biases: local feature extraction from convolutions and broader contextual reasoning from attention or sequence modeling modules. In many studies, hybrid designs report improvements in tumor detection, segmentation accuracy, and boundary precision compared to single-paradigm architectures.

Nevertheless, the reported gains are often incremental and dataset-dependent [30,47]. The combination of multiple modules increases model capacity and risk of overfitting, particularly on small or homogeneous datasets. Careful integration and tuning are required to prevent performance degradation due to conflicting feature representations or unstable training dynamics. Additionally, cross-dataset generalization remains a persistent challenge, suggesting that architectural hybridization alone does not resolve the fundamental issues of data scarcity, domain shift, and class imbalance in liver tumor segmentation.

### 5.2. Pancreas Tumor Segmentation

Pancreas tumor segmentation is considerably more challenging than liver tumor segmentation due to the pancreas’ small size, irregular geometry, weak boundary contrast, and high anatomical variability across patients. Tumor regions often exhibit subtle intensity differences from surrounding parenchyma, and severe class imbalance further complicates learning. From a computer vision standpoint, pancreas tumor segmentation demands precise localization, robust boundary modeling, and strong volumetric consistency under limited data conditions. Various approaches related to pancreas tumor segmentation are summarized in Table 4.

#### 5.2.1. Baseline U-Net and Cascaded CNN Architectures

Baseline convolutional architectures, particularly U-Net variants and cascaded CNN frameworks, remain widely adopted for pancreas and tumor segmentation [80,88,92]. These models typically perform pancreas localization in a first stage, followed by tumor refinement within a cropped region of interest. Such cascaded strategies improve training stability and reduce search space complexity, resulting in reliable pancreas Dice scores and moderate tumor segmentation performance.

However, despite stable convergence and straightforward deployment, these approaches rely primarily on local convolutional receptive fields. As a consequence, tumor Dice scores remain comparatively low, particularly for small or infiltrative lesions [85]. The limited modeling of long-range spatial dependencies restricts their ability to capture global anatomical context, which is crucial for distinguishing tumor tissue from surrounding structures.

#### 5.2.2. Attention and Multiscale CNN Models

To address scale variability and boundary ambiguity, attention mechanisms and multiscale feature aggregation strategies have been integrated into CNN-based architectures. These models enhance scale awareness by fusing features at different resolutions and applying channel or spatial attention to highlight tumor-relevant regions [81,83,87].

Empirically, attention-enhanced models improve Dice scores relative to plain U-Net baselines, particularly in boundary-sensitive cases. They better preserve fine structural details and improve tumor localization within complex abdominal backgrounds. Nevertheless, these methods remain fundamentally convolutional, and their receptive fields are still dominated by local feature extraction. Consequently, improvements in global volumetric consistency are often incremental rather than transformative.

#### 5.2.3. Transformer-Based Models

Recent work explores hybrid architectures that combine convolutional encoders with transformer-based or state-space (e.g., Mamba-inspired) modules. These models [82,108,109] aim to capture long-range dependencies and improve volumetric coherence across slices. By integrating global self-attention or sequence modeling mechanisms, they address one of the principal weaknesses of CNN-only frameworks.

Hybrid models typically report improved Dice and IoU metrics compared to baseline CNNs, particularly in maintaining tumor continuity across slices. They demonstrate enhanced global anatomical reasoning and better suppression of false positives. However, these gains come at the cost of increased computational complexity and memory requirements. Furthermore, pancreas tumor segmentation lacks large, standardized benchmarking datasets, making cross-study comparisons less consistent than in liver segmentation research.

#### 5.2.4. Clinical Reasoning and Diffusion-Refinement Models

Some approaches incorporate clinical priors or diffusion-based refinement strategies to improve robustness in multi-center settings. These models [90,110,111] refine coarse tumor predictions using iterative denoising or reasoning modules designed to enhance structural plausibility and reduce domain shift effects. Such methods demonstrate improved robustness across heterogeneous imaging protocols and report competitive Dice scores in cross-institutional evaluations. However, they involve complex training procedures and have not yet achieved widespread adoption within the broader computer vision community.

## 6. Discussion

### 6.1. Datasets

Standardized benchmarks and large multi-institutional datasets play a critical role in addressing the substantial heterogeneity present in medical imaging. Differences in scanner manufacturers, acquisition protocols, reconstruction kernels, contrast timing, and patient populations often lead to domain shifts that degrade model performance when deployed outside the training environment. Multi-institutional datasets mitigate these issues by capturing a broader distribution of imaging characteristics, enabling models to learn representations that are less sensitive to site-specific variations and more robust across clinical settings.

In parallel, standardized benchmarking frameworks improve evaluation consistency by enforcing uniform metrics, predefined train–test splits, and reproducible evaluation pipelines. Public challenges such as the LiTS challenge provide curated, multi-center CT datasets with harmonized annotations, allowing objective and comparable assessment of liver and tumor segmentation algorithms [20]. Similarly, the MSD includes liver and pancreas tasks sourced from multiple institutions, offering a controlled environment for evaluating model robustness under diverse imaging conditions [77]. These benchmarks reduce methodological variability and ensure that performance differences reflect genuine algorithmic improvements rather than inconsistencies in data handling or evaluation procedures.

Training and validating models on heterogeneous, multi-institutional datasets has been shown to improve cross-site generalization, reduce performance degradation on unseen scanners, and enhance stability across clinical workflows [112]. Exposure to diverse imaging domains also facilitates the development of domain-adaptation strategies, calibration techniques, and uncertainty-aware models—capabilities that are essential for safe and reliable clinical deployment. Consequently, the integration of standardized benchmarks with comprehensive multi-institutional datasets provides a rigorous foundation for developing segmentation models that are not only high-performing in research settings but also reproducible, generalizable, and clinically trustworthy.

### 6.2. Segmentation Approaches

Several strategies have been proposed to address the high computational cost and data dependency of transformer-based models. Hybrid CNN–Transformer architectures efficiently extract local features while modeling global context and reducing parameter complexity. Transfer learning and self-supervised pretraining can also improve performance when annotated datasets are limited. Furthermore, patch-based Transformer designs and lightweight attention modules reduce memory requirements, making these models more practical for medical imaging applications.

Recent work in medical image segmentation has increasingly focused on complex deep learning architectures that incorporate numerous modules, attention blocks, or hybrid components. While these designs may yield incremental performance gains, they often come at the cost of higher computational demands, longer training times, and reduced interpretability. For real-world clinical deployment, models must be efficient, transparent, and straightforward to integrate into existing hospital systems.

Lightweight hybrid CNN–Transformer architectures aim to balance local feature extraction and global contextual modeling by combining convolutional encoders with computationally efficient attention mechanisms. In many recent designs, CNN layers are used to capture fine-grained spatial features, while Transformer modules are introduced only in deeper network stages or bottleneck layers to model long-range dependencies. This design reduces the number of tokens processed by the Transformer, and therefore, limits computational overhead. To mitigate overfitting on relatively small medical imaging datasets, studies commonly employ regularization strategies, data augmentation, transfer learning, and multi-scale feature fusion. Such design choices allow hybrid architectures to retain the contextual benefits of Transformers while maintaining the efficiency and stability of convolutional networks.

Several studies that were reviewed reported liver Dice scores in the range of 0.96–0.97 when evaluated using widely accepted benchmark datasets, such as LiTS. This suggests that the performance of whole-liver segmentation has reached a practical threshold under standard experimental conditions. However, this observation should be interpreted cautiously within the context of a systematic literature review. Since many studies use the same datasets, evaluation metrics, and similar training protocols, the apparent convergence in reported performance could reflect the characteristics of the datasets and the methodological similarities rather than definitive evidence of architectural saturation. Therefore, improvements reported across studies should be considered in light of dataset dependence, variability in evaluation, and differences in experimental design.

Although the DSC is widely used for evaluating segmentation performance, it does not fully capture clinically relevant aspects of tumor delineation. Complementary boundary-based metrics such as the HD and ASSD quantify surface deviations and are particularly important for assessing the accuracy of tumor margins, which directly affects surgical planning and radiotherapy dose calculations. Volumetric similarity metrics, including relative volume difference and volume overlap error, provide insight into agreement in tumor burden estimation—an essential factor for staging, treatment response assessment, and eligibility for certain interventions. Incorporating a composite set of overlap, boundary, and volumetric metrics therefore enables a more comprehensive evaluation of segmentation performance and better reflects the requirements of real-world clinical decision-making.

The reported DSC values should be interpreted in the context of the datasets and evaluation protocols used in each study. Differences in imaging modality, annotation quality, and patient composition across datasets can substantially affect achievable performance. Likewise, variations in validation strategies, such as k-fold cross-validation, fixed train–test splits, or challenge-defined evaluation servers, lead to non-comparable Dice ranges.

Many studies report only mean segmentation metrics without accompanying confidence intervals or statistical significance testing, which limits the reliability of performance comparisons and obscures the variability inherent in model predictions. Confidence intervals provide essential information about the stability and uncertainty of reported metrics, while statistical tests allow for more rigorous assessment of whether observed performance differences between models are meaningful rather than attributable to sampling variability.

Moreover, inconsistencies in validation strategies further complicate cross-study comparison. Variations in cross-validation protocols, fold definitions, patient-level splits, and the use of internal versus external test sets can lead to substantial differences in reported performance. Standardizing validation procedures, such as adopting patient-level k-fold cross-validation, clearly separating training and test cohorts, and reporting results across all folds rather than a single split, would greatly improve reproducibility and comparability across studies. Incorporating these statistical and methodological practices is essential for generating robust evidence and ensuring that segmentation models are evaluated in a manner that supports reliable clinical translation.

Pancreatic tumor segmentation remains particularly challenging due to the small lesion size, irregular geometry, and weak contrast relative to surrounding tissues. To address these issues, recent studies have explored a range of specialized loss functions.

- Dice loss directly optimizes region overlap and mitigates severe class imbalance between tumor and background.
- Focal loss down-weights easy examples and emphasizes hard-to-detect voxels, improving sensitivity to small or low-contrast tumors.
- Boundary-aware losses incorporate spatial distance maps to penalize deviations along tumor borders, which is particularly beneficial for lesions with irregular or poorly defined edges.
- Topology-aware losses have been introduced recently to preserve structural connectivity and enforce anatomically plausible predictions in complex abdominal regions.

Alongside loss-function design, data augmentation plays a critical role in improving robustness. Common strategies include geometric transformations (rotation, scaling, flipping), elastic deformations to simulate anatomical variability, and intensity-based augmentations that mimic contrast variations across scanners and acquisition protocols. These augmentations increase training diversity and help models generalize to the heterogeneous appearance of pancreatic tumors.

In addition, improving segmentation accuracy for small, low-contrast, and irregularly shaped tumors remains a critical challenge. Future research could explore architectural modifications that enhance both local boundary sensitivity and global contextual reasoning within U-Net based frameworks. In particular, attention-guided feature refinement, multi-scale feature aggregation, and hybrid CNN–Transformer architectures may improve the representation of subtle tumor structures that are often missed by conventional encoder–decoder networks. Incorporating deep supervision, boundary-aware loss functions, and feature pyramid structures may further strengthen sensitivity to small lesions and complex tumor morphologies. Beyond architectural improvements, advanced training strategies, including curriculum learning, hard example mining, and self-supervised pretraining, may help models learn more robust feature representations when annotated datasets are limited. Collectively, these approaches offer promising directions for overcoming the persistent limitations of current U-Net variants and improving the robustness and clinical applicability of hepatopancreatic tumor segmentation systems.

### 6.3. Clinical Relevance

Automated tumor segmentation significantly supports clinical practices by reducing the time needed for manual delineation and improving the reproducibility of imaging analysis. In current clinical practice, tumor boundaries are often outlined manually by radiologists, a process that is time-consuming and subject to interobserver variability. Deep learning-based segmentation systems can assist clinicians by providing fast, consistent delineations of tumor regions. Accurate segmentation is crucial for applications such as surgical planning, radiotherapy targeting, and monitoring treatment response because precise estimation of tumor boundaries and volumes directly influences treatment decisions and outcome assessment.

Despite these potential benefits, several challenges still limit the adoption of these systems in clinical practice. Models trained on limited or single-institution datasets may have difficulty generalizing across hospitals due to variations in imaging protocols, scanner types, and patient populations. Additionally, robust validation across diverse, multi-institutional datasets is necessary to ensure reliability and clinical safety. Improved generalization techniques, standardized evaluation protocols, and larger collaborative datasets are essential for translating automated tumor segmentation methods into routine clinical practice.

## 7. Challenges

To provide a structured overview of the challenges, Table 5 summarizes the methodological issues and structural limitations identified in liver and pancreas tumor segmentation. Methodological issues refer to biases that arise from the design, data, and evaluation of the reviewed studies, including dataset size, annotation variability, architectural choices, and inconsistent evaluation protocols. Structural limitations represent broader barriers inherent to the discipline, such as limited availability of annotated datasets, variability in imaging protocols, inherent anatomical complexity, and computational constraints. This taxonomy highlights the sources of bias affecting segmentation performance and facilitates a clear distinction between study-specific challenges and discipline-level limitations.

A systematic analysis of recent deep learning approaches reveals persistent challenges that limit segmentation accuracy, robustness, and clinical translation in both liver and pancreas tumor segmentation. These limitations arise from anatomical complexity, tumor heterogeneity, architectural constraints, computational burden, and dataset variability. They are consistently observed across widely used benchmarks such as LiTS 2017, 3DIRCADb-01, MSD Pancreas, NIH Pancreas-CT, MSD Liver, and CHAOS. For clarity, these challenges are grouped into four main categories.

### 7.1. Memory, Context, and Anatomical Complexity

Liver and pancreas tumor segmentation is typically performed on high-resolution 3D CT or MRI volumes, which substantially increase GPU memory consumption and computational cost. Training is often restricted to small batch sizes, leading to optimization instability and slower convergence. Patch-based strategies are frequently adopted to mitigate memory limitations; however, they compromise global spatial context and may reduce localization accuracy, particularly for small or spatially dispersed tumors.

Modeling volumetric continuity across slices remains computationally intensive. Learning meaningful three-dimensional anatomical relationships is especially challenging in the pancreas, whose shape is highly irregular and varies significantly across patients. In addition, both the liver and pancreas are surrounded by tissues with similar intensity distributions, making boundary discrimination difficult. Imaging artifacts, noise, low contrast, and intensity inhomogeneity further degrade feature representation and increase boundary ambiguity.

### 7.2. Tumor Variability and Boundary Uncertainty

Tumors in both organs exhibit substantial variability in size, morphology, texture, and spatial distribution. Liver tumors may appear as multiple dispersed lesions, while pancreatic tumors are often small, infiltrative, and poorly contrasted against surrounding tissue. Such variability complicates consistent feature learning and increases sensitivity to model initialization and training conditions.

Low contrast between tumor and parenchyma frequently results in indistinct or discontinuous boundaries. Small lesions or tumors partially occluded by adjacent anatomical structures are particularly prone to under-segmentation or missed detection. Although multi-scale fusion and attention mechanisms improve sensitivity, they increase computational demands and do not fully resolve boundary ambiguity.

### 7.3. Architectural and Feature Representation Limitations

Segmentation networks must simultaneously preserve fine-grained boundary details and capture long-range contextual dependencies. Conventional CNN-based architectures provide strong local feature extraction but are limited in modeling global relationships. Transformer-based and hybrid architectures address this limitation by incorporating attention mechanisms; however, in 3D settings they significantly increase memory consumption and computational complexity.

Increasing architectural depth and structural complexity often introduces optimization difficulties, including training instability and diminishing returns in performance gains. Maintaining boundary precision while learning high-level semantic representations remains particularly challenging in pancreas tumor segmentation, where anatomical variability is greater and tumor appearance is less distinct. Thus, balancing global reasoning, multiscale representation, and computational efficiency remains an unresolved design trade-off.

### 7.4. Dataset Variability and Generalization

Dataset heterogeneity is a major barrier to robust clinical deployment. Public benchmarks differ substantially in acquisition protocols, scanner parameters, contrast phases, annotation standards, and tumor distributions. Cross-modality datasets introduce additional variability between CT and MRI intensity characteristics. Furthermore, pancreas datasets are generally smaller and exhibit greater inter-patient anatomical variability than liver datasets, exacerbating overfitting risks.

Manual annotations of volumetric scans are labor-intensive and subject to inter-observer variability, introducing label noise. Consequently, models trained on a single dataset often exhibit reduced performance when evaluated on external data, highlighting persistent generalization limitations.

## 8. Conclusions

This systematic review provides a comprehensive overview of deep learning approaches for hepatopancreatic tumor segmentation published between 2021 and 2026, emphasizing architectural innovations, training paradigms, and dataset utilization from a computer vision standpoint. Across the literature, U-Net and its variants remain the dominant baseline, achieving near-saturated liver segmentation performance but often struggling with tumor delineation due to small lesion size, low contrast, and heterogeneous morphology. Encoder–decoder refinements offer incremental improvements, while transformer-based and hybrid CNN–Transformer architectures improve global context modeling and small lesion detection, though at the cost of increased computational complexity and sensitivity to dataset scale.

Compared to liver tumor segmentation, pancreas tumor segmentation remains less mature and more performance-constrained. Baseline CNN models provide stable pancreas localization but struggle with small and heterogeneous tumors. Attention mechanisms and multiscale fusion improve local feature representation, while hybrid CNN–Transformer architectures enhance global consistency at increased computational cost.

Overall, while deep learning has significantly advanced hepatopancreatic tumor segmentation, fundamental challenges, particularly small lesion detection, robust boundary delineation, and generalization across heterogeneous datasets, remain open. Future research should focus on integrating efficient global context modeling, domain-adaptive training strategies, and robust normalization techniques to mitigate domain shift caused by variations in imaging protocols, scanner types, and institutional datasets. In addition, federated learning frameworks offer a promising direction for enabling collaborative multi-institutional model training while preserving patient privacy, thereby improving dataset diversity and model robustness. The integration of these approaches, together with semi-supervised or weakly supervised learning strategies, may support the development of scalable, accurate, and clinically applicable hepatopancreatic tumor segmentation frameworks.

## Figures and Tables

**Table 1:** List of publicly available datasets focused on liver and/or pancreas segmentation that were used in the included studies.

Dataset	Organ	Imaging Modality	Size (Cases)	Description	Ref.
LiTS 2017 [20]	Liver	CT	201	2 categories of liver and tumor segmentation	[21,22,23,24,25,26,27,28,29,30,31,32,33,34,35,36,37,38,39,40,41,42,43,44,45,46,47,48,49,50,51,52,53,54,55,56,57,58,59,60,61,62,63,64,65,66,67,68,69,70,71]
3DIRCADb-01 [72]	Liver	CT	22	40 categories of abdominal organ and tumor segmentation	[21,23,26,32,33,34,35,40,41,45,48,49,54,67,68,73,74,75,76]
MSD Pancreas [77]	Pancreas	CT	420	2 categories of pancreas and tumor segmentation	[78,79,80,81,82,83,84,85,86,87,88]
NIH Pancreas-CT [89]	Pancreas	CT	82	1 category of healthy pancreas segmentation	[83,86,88,90,91,92]
MSD Liver [77]	Liver	CT	131	Liver and tumor segmentation	[34,65,85]
CHAOS [93]	Liver	CT & MRI	40	4 categories of abdominal organ segmentation	[23,27,35]
BTCV [94]	Liver, Pancreas	CT	50	13 categories of abdominal organ segmentation	[27,82]
DLDS [95]	Liver	MRI	105	Liver disease classification and segmentation	[96]
ATLAS [97]	Liver	CT & MRI	90	Liver and HCC tumor segmentation	[96]
Radiopaedia ^1^	Liver	CT	Varies	Open-access 3D CT cases with liver annotations	[22]
MedSeg [98]	Liver	CT	50	Manual segmentations of liver segments 1–8	[30]
HCC-TACE-Seg [99]	Liver	CT	105	4 categories of liver and tumor segmentation	[100]
FLARE 2021 [101]	Liver, Pancreas	CT	511	4 categories of abdominal organ segmentation	[90]
AbdomenAtlas Mini [102]	Liver, Pancreas	CT	5195	9 categories of abdominal organ segmentation	[103]

^1^ https://radiopaedia.org/.

**Table 2:** Summary of evaluation metrics for tumor segmentation.

Metric	Ideal Value	Description	Clinical
Overlap-based Metrics			
DSC	1.0	Spatial overlap between regions.	Standard for general accuracy.
IoU	1.0	Intersection over Union.	Penalizes errors more strictly than DSC.
VOE	0%	Volumetric error percentage.	Direct measure of volume disagreement.
Distance-based Metrics			
HD/HD95	0.0	Max boundary distance.	Critical for surgical precision and margins.
ASSD	0.0	Mean symmetric surface distance.	Evaluates average boundary alignment. Less sensitive to outliers than HD.
Statistical Measures			
Accuracy	1.0	Global pixel correctness.	Use with caution on imbalanced data.
Precision	1.0	Reliability of positives.	Reduces over-diagnosis (False Positives).
Sensitivity	1.0	Detection of positives.	Reduces missed pathology (False Negatives).
F1-Score	1.0	Harmonic mean of P & S.	Balanced metric for imbalanced classes.
AUC	1.0	Discriminative power.	Robust performance across thresholds.

**Table 3:** Segmentation models applied to liver tumor.

Model Type	Strengths	Limitations	Ref.
U-Net and Variants	Strong baseline. The skip in U-Net connections transfer high-resolution spatial information directly. U-Net works well for boundary delineation and tumor segmentation, especially on small medical imaging datasets. Stable performance.	Tumors that are very small, low contrast, deformable, or irregularly shaped may be under-segmented or missed. Limited long-range dependency modeling.	[21,22,23,28,30,31,32,36,38,42,43,44,46,48,50,53,55,61,67,68,69,71,74,76,104,105]
Encoder Decoder Networks	Good feature extraction. Avoids blurred or coarse segmentation masks, which is critical for surgical planning.	May miss small tumors with low contrast against liver tissue. Noise, motion artifacts, or variable intensity in CT/MRI scans can degrade accuracy. Limited global context modelling.	[24,28,37,40,49,56,59,66,100]
Transformer-based Models	Better global feature modeling. Effective for small lesion segmentation. Unlike standard CNNs or U-Nets, transformers don’t rely solely on local receptive fields. Their Self-attention module can relate distant regions to improve tumor localization.	Requires large data. Higher computational complexity.	[24,34,35,41,50,54,58,60,62,96,106]
Optimization/Clinical Reasoning	These models typically employ lightweight architectures that achieve higher accuracy while minimizing the risk of overfitting. Improves tumor boundary precision.	Sensitive to initial segmentation quality. Optimized models still struggle if the training dataset is too small or not diverse. Hyperparameter and architecture optimization require expert knowledge and time.	[22,34,45,51,57,63,100]
Attention-Based Networks	They are excellent at focusing on important regions, capturing complex tumor shapes, and reducing noise.	They come with higher computational cost and require careful design and sufficient data.	[25,26,31,32,33,36,38,39,46,48,53,55,61,62,70]
Mask-RCNN	Ideal when both tumor detection and precise segmentation are needed, especially for multiple or irregular tumors.	Lower performance than dedicated segmentation networks. Two-stage pipeline and mask head increase memory and inference time, especially for high-resolution CT/MRI scans.	[44,52,64,75]
Graph CNN	Graph CNNs capture Non-Euclidean relationships. Therefore, they are powerful for modeling complex spatial relationships and irregular tumor shapes.	Converting images to graphs (nodes + edges) can be computationally expensive.	[39,43,51]
Hybrid CNN Models	Combines multiple architectures to improve tumor detection, segmentation, and boundary precision.	They can overfit small datasets. Thus, they require careful integration and tuning of different modules to avoid performance degradation.	[24,25,26,27,29,30,33,34,35,47,96]

**Table 4:** Segmentation models applied to pancreas tumor.

Model Type	Strengths	Limitations	Ref.
U-Net and Variants	Stable training. Simple deployment. Good pancreas localization.	Limited global context. Tumor Dice remains low.	[79,80,85,88,92]
Attention-based Networks	Improved boundary and scale awareness.	Still mainly local receptive fields.	[81,83,87]
Transformer-based Models	Capture long-range dependencies. Better volumetric consistency.	Computationally complex. Limited standardized benchmarks.	[82,108,109]
Clinical reasoning/Optimization	Improved multi-center robustness.	Complex training. Limited adoption.	[90,110,111]

**Table 5:** Methodological and structural challenges in liver and pancreas tumor segmentation.

Category	Subcategory/Bias Type	Description
Methodological Issues	Data-related	Small datasets. Class imbalance. Inter-observer annotation variability. Lack of external validation.
	Model-related	Architectural choices that trade off boundary precision for global reasoning. Reliance on patch-based or 2D approximations reducing contextual understanding. Limited generalization across anatomical variability.
	Evaluation-related	Inconsistent evaluation metrics. Over-reliance on AUC in imbalanced datasets. Lack of standardized evaluation protocols.
Structural Limitations of the Discipline	Dataset availability	Limited availability of large, annotated 3D medical imaging datasets.
	Acquisition variability	Variability in imaging protocols, scanner types, contrast phases, and cross-modality intensity characteristics.
	Anatomical & tumor complexity	Inherent anatomical variability and tumor heterogeneity that are difficult to model consistently.
	Computational constraints	High memory and computational demands for 3D volumetric segmentation, limiting adoption of more advanced architectures.

## Data Availability

The data presented in this study are available on request from the corresponding author, as no new data were created in this study and all data were obtained from published literature.

## Appendix A

### Appendix A.1

The PICO(S) framework for the literature search is listed in Table A1.

**Table A1:** PICO(S) framework.

Component	Description
Population (P)	Patients with liver tumors or pancreatic tumors imaged using CT or MRI datasets.
Intervention (I)	Deep learning-based tumor segmentation methods applied to liver or pancreas imaging.
Comparison (C)	Traditional image processing approaches, conventional machine learning methods, or alternative deep learning architectures when reported.
Outcomes (O)	Segmentation performance metrics such as Dice Similarity Coefficient (DSC), Intersection over Union (IoU), precision, recall, F1-score, and AUC.
Study Design (S)	Peer-reviewed studies reporting the development or evaluation of deep learning models for tumor segmentation in liver or pancreas imaging.

### Appendix A.2

The list of studies included in the review is listed in Table A2.

**Table A2:** List of studies included in the systematic literature review.

Ref. ID	Year	Paper Title
[21]	2026	TCA-Net: Liver tumor segmentation based on a new contextual feature encoding module and attention mechanism
[96]	2026	Enhanced 3D tumor synthesis and segmentation framework using multiscale diffusion and hybrid SAM-Swin models across diverse anatomical datasets
[22]	2025	A ResNet-50–UNet Hybrid with Whale Optimization Algorithm for Accurate Liver Tumor Segmentation
[23]	2025	A novel unified Inception-U-Net hybrid gravitational optimization model (UIGO) incorporating automated medical image segmentation and feature selection for liver tumor detection
[24]	2025	DEDSWIN-Net: Dual Encoder Dilated Convolution and Swin Transformer Network for the Classification of Liver CT Images
[25]	2025	FasNet: a hybrid deep learning model with attention mechanisms and uncertainty estimation for liver tumor segmentation on LiTS17
[26]	2025	A hybrid attention-based deep learning model for segmentation of livers and liver tumors from CT scans
[113]	2025	Geometric deep learning adapted to prediction of liver resection zone
[27]	2025	Intelligent Tumor Synthesis Based on Medical Image Knowledge for Liver Tumor Segmentation
[28]	2025	RIS-UNet: A Multi-Level Hierarchical Framework for Liver Tumor Segmentation in CT Images
[29]	2025	Automatic liver tumor segmentation based on improved Yolo-v5 and B-spline level set
[30]	2025	Effective Tumor Annotation for Automated Diagnosis of Liver Cancer
[31]	2025	Enhancing lesion detection in liver and kidney CT scans via lesion mask selection from two models: A main model and a model focused on small lesions
[32]	2025	GLCFANet: Liver Tumor Segmentation with Global and Local Information Cross-Fusion Attention
[33]	2025	DGA-Net: a dual-branch group aggregation network for liver tumor segmentation in medical images
[34]	2025	QCNN-Swin-UNet: Quantum Convolutional Neural Network Integrated with Optimized Swin-UNet for Efficient Liver Tumor Segmentation and Classification on Edge Devices
[35]	2025	ICT-Net: An Integrated Convolution and Transformer-Based Network for Complex Liver and Liver Tumor Region Segmentation
[36]	2025	A Deep Learning Approach for Automatic Liver Segmentation: Attention U-Net with ASPP and CBAM Integration
[100]	2025	SmoothSegNet: A global-local framework for liver tumor segmentation with clinical knowledge-informed label smoothing
[104]	2025	Multi-Phase Attention for Early Stage Hepatocellular Carcinoma Segmentation from Contrast Enhanced Liver MRI
[37]	2025	Patch-Based Deep-Learning Model With Limited Training Dataset for Liver Tumor Segmentation in Contrast-Enhanced Hepatic Computed Tomography
[108]	2025	DCM-Net: Dual-Encoder CNN-Mamba Network with Cross-Branch Fusion for Robust Medical Image Segmentation
[78]	2025	MMPU-Net: A Parameter-Efficient Network for Fine-Stage of Pancreas and Pancreas-Tumor Segmentation on CT Scans
[79]	2025	LooBox: Loose-Box-Supervised 3D Tumor Segmentation with Self-Correcting Bidirectional Learning
[90]	2025	Segment Like A Doctor: Learning Reliable Clinical Thinking and Experience for Pancreas and Pancreatic Cancer Segmentation
[114]	2025	Enhancing Detection of Various Pancreatic Lesions on Endoscopic Ultrasound Through Artificial Intelligence: A Basis for Computer-Aided Detection Systems
[73]	2025	Automated 3D Visualization and Volume Estimation of Hepatic Structures for Treatment Planning of Hepatocellular Carcinoma
[80]	2025	Attention U-Net for Segmentation of Pancreatic Neuroendocrine Neoplasms in Computer Tomograph
[109]	2025	SMF-Net: Semantic-Guided Multimodal Fusion Network for Precise Pancreatic Tumor Segmentation in Medical CT Image
[81]	2025	Scale-View Co-Awareness Framework for Simultaneous Segmentation of Pancreas and Tumors
[82]	2025	3D-SCUMamba: An Abdominal Tumor Segmentation Model
[103]	2025	Efficient Human-in-the-Loop Pancreatic Tumor Annotation via Large-Scale Pre-Trained Model with Adaptive Post-Processing
[38]	2025	MMEFU-Net: A Mamba-Guided Multi-Encoder Fusion U-Net for Tumor Segmentation in CT Images
[39]	2025	Ultra-Lightweight Network for Medical Image Segmentation Inspired by Bio-Visual Interaction
[115]	2024	MRI radiomics based on deep learning automated segmentation to predict early recurrence of hepatocellular carcinoma
[74]	2024	Real-Time Liver Tumor Detection with a Multi-Class Ensemble Deep Learning Framework
[40]	2024	A dual-encoder double concatenation Y-shape network for precise volumetric liver and lesion segmentation
[75]	2024	A Novel Liver Tumor segmentation of Adverse Propagation Advanced Swin Transformer Network with Mask region-based convolutional neural networks
[41]	2024	AD-DUNet: A dual-branch encoder approach by combining axial Transformer with cascaded dilated convolutions for liver and hepatic tumor segmentation
[42]	2024	VU-NET: An explainable AI approach for liver segmentation
[43]	2024	Optimized Liver Tumor Segmentation: Integrating U-Net with Graph Cut Algorithms
[44]	2024	SEU2-Net: multi-scale U2-Net with SE attention mechanism for liver occupying lesion CT image segmentation
[45]	2024	A three-path network with multi-scale selective feature fusion, edge-inspiring and edge-guiding for liver tumor segmentation
[46]	2024	Deep learning based automatic internal gross target volume delineation from 4D-CT of hepatocellular carcinoma patients
[47]	2024	FasNet: An Advanced Deep Learning Model for Liver Tumor Segmentation
[83]	2024	Attention-Enhanced Multiscale Feature Fusion Network for Pancreas and Tumor Segmentation
[84]	2024	Multi-Scale Adversarial Learning with Difficult Region Supervision Learning Models for Primary Tumor Segmentation
[85]	2024	A Deep Learning-Based Interactive Medical Image Segmentation Framework with Sequential Memory
[86]	2024	IDRCNN: A Novel Deep Learning Network Model for Pancreatic Ductal Adenocarcinoma Detection on Computed Tomography
[116]	2024	Weakly Supervised Large-Scale Pancreatic Cancer Detection Using Multi-Instance Learning
[48]	2023	MANet: a multi-attention network for automatic liver tumor segmentation in computed tomography (CT) imaging
[49]	2023	Context fusion network with multi-scale-aware skip connection and twin-split attention for liver tumor segmentation
[50]	2023	Joint liver and hepatic lesion segmentation in MRI using a hybrid CNN with transformer layers
[51]	2023	Automatic Liver Tumor Segmentation from CT Images Using Graph Convolutional Network
[52]	2023	Slice-Fusion: Reducing False Positives in Liver Tumor Detection for Mask R-CNN
[53]	2023	SUNet++: A Deep Network with Channel Attention for Small-Scale Object Segmentation on 3D Medical Images
[54]	2023	DHT-Net: Dynamic Hierarchical Transformer Network for Liver and Tumor Segmentation
[55]	2023	Eres-UNet++: Liver CT image segmentation based on high-efficiency channel attention and Res-UNet++
[56]	2023	CEDRNN: A Convolutional Encoder-Decoder Residual Neural Network for Liver Tumour Segmentation
[57]	2023	Detection of Liver Tumour Using Deep Learning Based Segmentation with Coot Extreme Learning Model
[91]	2023	VGG16 Feature Extractor with Extreme Gradient Boost Classifier for Pancreas Cancer Prediction
[92]	2023	U-Net-Based Pancreas Tumor Segmentation from Abdominal CT Images
[87]	2023	Temperature Guided Network for 3D Joint Segmentation of the Pancreas and Tumors
[59]	2023	Advanced Learning-Based Segmentation of Liver and Tumor 3D Images for Early Disease Diagnosis
[60]	2023	SCTransNet: 3D Medical Image Segmentation Model Based on the Fusion of CNN and Transformer
[61]	2023	Modif-SegUnet: Innovatively Advancing Liver Cancer Diagnosis and Treatment through Efficient and Meaningful Segmentation of 3D Medical Images
[62]	2023	ACF-TransUNet: Attention-based Coarse-Fine Transformer U-Net for Automatic Liver Tumor Segmentation in CT Images
[63]	2023	Boundary Optimization Scheme for Liver Tumors from CT Images
[106]	2023	Deep Learning-powered Multiple-object Segmentation for CAD
[58]	2022	Towards Optimal Patch Size in Vision Transformers for Tumor Segmentation
[105]	2022	Deep Learning Algorithms in the Automatic Segmentation of Liver Lesions in Ultrasound Investigations
[64]	2022	Data augmentation based on multiple oversampling fusion for medical image segmentation
[65]	2022	Learning From Synthetic CT Images via Test-Time Training for Liver Tumor Segmentation
[76]	2022	A computer-aided diagnostic system for liver tumor detection using modified U-Net architecture
[66]	2022	DTSC-Net: Semi-supervised 3D Biomedical Image Segmentation through Dual-Teacher Simplified Consistency
[67]	2022	HFRU-Net: High-Level Feature Fusion and Recalibration UNet for Automatic Liver and Tumor Segmentation in CT Images
[88]	2022	AX-Unet: A Deep Learning Framework for Image Segmentation to Assist Pancreatic Tumor Diagnosis
[117]	2022	Improved Pancreatic Tumor Detection by Utilizing Clinically-Relevant Secondary Features
[68]	2021	X-Net: Multi-branch UNet-like network for liver and tumor segmentation from 3D abdominal CT scans
[69]	2021	A Multi-Scale and Multi-Resolution Approach for Liver Tumor Segmentation in CT Scans
[70]	2021	Advanced Deep Learning Approach to Automatically Segment Malignant Tumors and Ablation Zone in the Liver With Contrast-Enhanced CT
[71]	2021	CT Segmentation of Liver and Tumors Fused Multi-Scale Features
[110]	2021	Diminishing Uncertainty within the Training Pool: Active Learning for Medical Image Segmentation
[111]	2021	Multi-Task Federated Learning for Heterogeneous Pancreas Segmentation
